# Protection of our Future Generation: Use of Face Masks in Children amidst COVID-19 Era

**DOI:** 10.31729/jnma.6705

**Published:** 2021-08-31

**Authors:** Mallika Gyawali

**Affiliations:** 1 Tranquility Hospital And Research Center, Lalitpur, Nepal

**Keywords:** *child*, *COVID-19*, *hand disinfection*, *masks*

## Abstract

During the Coronavirus disease-19 (COVID-19) crisis, the universal use of face masks in addition to hand washing and practicing safe distancing is recommended worldwide. Children, like adults, also get infected through direct contact or airborne droplets. Face masks efficiently prevent respiratory droplet transmissions. However, asking children to wear face masks can be challenging and may have potential advantages and disadvantages. In this viewpoint, we discuss the effectiveness of face mask usage in children and its associated challenges, myths, advantages, and disadvantages.

## INTRODUCTION

In children, infection with the virus Severe Acute Respiratory Syndrome Coronavirus-2 (SARS-CoV-2) is asymptomatic or mild in nature.^1^ Some can develop life threatening symptoms or a severe multisystem inflammatory syndrome.^2^ Along with wearing masks, children should be taught appropriate hand washing with soaps and physical distancing that are proven techniques preventing the viral spread.^3^ While using any type of mask, a cloth mask or a surgical mask, ensuring the right fit is crucial.^4^ However, parents are usually concerned about their children wearing masks.^5^ Therefore, this article highlights the advantages and importance of using masks in children.

## GUIDELINES FOR WEARING A MASK

**Table 1:** Recent CDC guidelines for wearing a mask.^6^

Mask should completely cover the nose and mouth and fit snugly against the sides of the face without gaps. Children aged two and older should wear masks in public settings and when around people who don’t live in their household. Masks should not be worn by children under two years of age or anyone who has trouble breathing or is unconscious or otherwise unable to remove a facecovering on their own. Everyone aged 12 or older is now recommended to get a COVID-19 vaccination and fully vaccinated ones can resume their daily activities without wearing a mask or physically distancing.^7^

## TYPES OF FACE MASKS TO BE WORN BY CHILDREN

The World Health Organization (WHO) advises people, inclusive of children, wear fabric or non-medical masks, which needs to be washed after each use.^8^ Whatever the type of masks, medical or surgical, it should only be used if children have some concerning health conditions, have symptoms or signs of or confirmed coronavirus disease 2019 (COVID-19), or are in the vicinity of someone who is sick.^9^

## HOW TO MOTIVATE CHILDREN TO WEAR MASKS?

There are innovative ways to make mask-wearing an amusing experience for children. Use simple language and explain how masks keep everyone safe. Introduce masks by wearing one yourself or letting one of their favorite stuffed animals wear one or showing them pictures of their favorite cartoon character in masks. Help your child choose their masks or put stickers or messages to decorate disposable masks. Let your child know not wearing masks in public isn't an option and try rewarding their mask-wearing. Wearing a mask is a new behavior, so be calm and boost this habit. When parents are well aware of the significance of masking, it will be easier to convince children to wear a mask and prevent viral contamination.^10^

## MYTHS ABOUT FACE MASK USE IN CHILDREN

Many parents are skeptical about the use of face masks on their children as there are various ongoing myths about masking in children.^11^ A few common myths include:

Myth: Masks make it harder to breathe.

Fact: As shown by a cohort study in Italy, the use of masks is not associated with a change in respiratory function with no episodes of oxygen desaturation or respiratory distress.^12^ Cloth masks are made of breathable material that allows enough oxygen transport.

Myth: Masks can trap the carbon dioxide we normally breathe out due to inadequate gas exchange.

Fact: Properly fitted masks allow adequate airflow. A study in the USA has shown that no gas exchange abnormalities have been observed with the usage of surgical masks.^13^ However, children below two years should not wear masks as they can't remove them by themselves and there are safety concerns.

Myth: Masks shut down the immune system.

Fact: Masks don't weaken the immune system; instead they prevent the spread of infection from person to person by acting as a physical barrier to the virus and they also prevent cooling of the nasal mucosa that can increase viral survival.^14^

Myth: There is no adequate data supporting mask usage.

Fact: A systematic review published in the Lancet journal showed masks, in general, are associated with a large reduction in risk of infection from SARS-CoV-2 and Middle East respiratory syndrome coronavirus 2 (MERS-CoV-2).^3,15^ Masking should be done in addition to practicing proper sanitation habits.

## ADVANTAGES OF WEARING A MASK IN CHILDREN

Globally, wearing masks has been one of the most competent preventive measures that can protect children by reducing the spread of virus-laden respiratory droplets. Masks can also provide protection against other various respiratory viruses like Influenza or Rhinovirus.^16^ Furthermore, masks can protect kids from dirt and allergens, keep their noses warm in winter, and keep bad smells away.

## DISADVANTAGES OF WEARING A MASK IN CHILDREN

Finding a mask that is the right fit for a child is pretty difficult. Masks can also hinder verbal conversations. Using face masks can replace the focus from other important measures such as distancing, hand washing, and staying home when feeling ill. Children can also keep on touching or adjusting their masks which can increase the viral load in their hands. Additionally, face masks hamper the mirroring of facial expressions which is an essential component of a teacher-student relationship.

## CHALLENGES OF FACE MASK USE IN CHILDREN

Finding an appropriately sized mask in the market for children is pretty difficult. Also, children are usually carefree and aren't worried about mask use and maintaining a distance while meeting their friends most of the time. Wearing facemasks in children can cause anxiety issues and seem scary occasionally. All these challenges can be handled with proper explanation and compensating proper behavior regarding masks.

## WAY FORWARD

At this time of the SARS-COV-2 pandemic, masking is a useful add-on to social distancing and hand hygiene practices. As children learn very quickly, they can adapt to masking with careful guidance from parents and teachers. Hence, even though there are various myths and disadvantages related to masking, wearing a proper mask by encouraging children will help prevent and control the spread of the coronavirus and make all our lives safer.
